# Supplementary material for: Population Genomics of Wall Lizards Reflects the Dynamic History of the Mediterranean Basin
Source: Mol Biol Evol. 2021 Oct 28;39(1):msab311. doi: 10.1093/molbev/msab311 (PMC8760935; doi:10.1093/molbev/msab311)
Supplement: msab311_Supplementary_Data [file msab311_supplementary_data.zip › supplementary materials.pdf]

Supplementary materials

Population genomics of wall lizards reflects the dynamic history of the Mediterranean Basin

Weizhao Yang, Nathalie Feiner, Daniele Salvi, Hanna Laakkonen, Daniel Jablonski, Catarina Pinho, Miguel A. Carretero, Roberto Sacchi, Marco A. L. Zuffi, Stefano Scali, Konstantinos Plavos, Panayiotis Pafilis, Nikos Poulakakis, Petros Lymberakis, David Jandzik, Ulrich Schulte, Fabien Aubret, Arnaud Badiane, Guillem Perez i de Lanuza, Javier Abalos, Geoffrey M. While, Tobias Uller

Supplementary materials contain:

6 supplementary tables

8 supplementary figures

Supplementary table S1 Information of sampling sites for RAD-Seq samples in this study.

Site	Longitude	Latitude
ALB2	19.633	42.3685
ALB4	19.668	40.9833
AQF	16.242	39.9874
BUL_MT	27.475	41.9706
BUL4	24.81	42.64
BUL5	25.99	41.76
BV	11.539	45.4106
CAR	-3.095	47.5746
CCR	14.404	44.9586
CDG	14.071	41.9954
CE	11.291	44.7315
COR	12.228	42.7033
CR	10.565	43.5748
CRO	17.053	43.3423
CRS	14.955	40.7835
CV	11.936	43.6981
CX	10.341	44.5331
EF_W	6.89	49.99
ENF	10.263	42.8277
FRM	13.741	43.172
GRU	9.1654	45.5682
KOP	13.727	45.5488
LE	9.6107	44.1686
MG	10.162	44.0122
MON	-1.8178	41.7902
MOR	-3.8295	40.8274
MP	11.163	44.0926
NL	8.4142	44.2063
NM	2.6762	48.2879
OLY	22.257	40.1155
OV	12.934	42.13
PDT	-3.8351	47.7966
PEN	-0.3339	40.2481
PI	9.5029	44.8198
PY	1.1052	42.8595
PZ	-0.8392	46.7844
RCM	14.35	41.4924
RO	12.544	41.8586

ROM2	21.68	44.73
ROM7	21.72	46.11
ROM9	23.96	45.42
SER_BO	20.655	43.4295
SER1	20.45	44.79
SER3	19.77	43.46
SER4	19.58	43.85
SER7	22.52	42.43
SLOVK2	17.073	48.1441
SLOVK4	19.483	49.1017
SR	7.7729	43.8183
ST	9.9046	44.0823
TRC	21.466	38.5896
TYG	22.362	36.9519
UR	12.636	43.7236
VI	10.263	43.8427
VTS	21.388	40.6138

Supplementary table S2 Information of sampling sites for WGS in this study

Site	Longitude	Latitude
AQF	16.24217	39.98745
Elba_east	10.360371	42.808416
Elba_west	10.169758	42.786448
GRC	21.726	39.1825
KOP	13.724	42.5488
LE	9.610654	44.16861
NL	8.414208	44.20628
NM	2.67624	48.28794
PDT	-3.83513	47.79656
PEN	-0.67	40.37
RCM	14.34966	41.49243
RO	12.54419	41.85863
SLOVK	17.075601	48.143695
SR	7.772901	43.81833
TK	27.42	41.97
TU	10.720143	43.544483

Supplementary table S3 *Podarcis* species and outgroup used in gene flow analysis

Sample_ID	Species	Note
Ab387.8	*Archaeolacerta bedriagae*	Outgroup
PB01	*Podarcis bocagei*	
DB28361	*Podarcis erhardii*	
2100-110	*Podarcis filfolensis*	
DB7019	*Podarcis hispanicus*	PHGAL lineage
DB28386	*Podarcis levendis*	
DB20801	*Podarcis melisellensis*	
DB13118	*Podarcis pityusensis*	
CAP3	*Podarcis siculus*	North lineage (N)
TRC3	*Podarcis siculus*	South lineage (S)
DB16305	*Podarcis tauricus*	
395A	*Podarcis tiliguerta*	Corsica lineage (C)
PF2	*Podarcis tiliguerta*	Sardinia lineage (S)
DB26449	*Podarcis waglerianus*	

Supplementary table S4 Results of D statistics for intraspecific comparison for *P. muralis* lineages

W	X	Y	D	Zscore
Southern Balkan	Northern Balkan	Central Italy	-0.048	-13.511
Western Europe (N)	Northern Balkan	Central Italy	-0.143	-31.426
Western Europe (N)	Southern Balkan	Central Italy	-0.1087	-22.867
Western Europe (S)	Northern Balkan	Central Italy	-0.1215	-24.86
Western Europe (S)	Southern Balkan	Central Italy	-0.086	-16.76
Western Europe (S)	Western Europe (N)	Central Italy	0.0365	10.546
Western Europe (S)	Western Europe (N)	Northern Balkan	0.0381	9.514
Central Italy	Northern Balkan	Southern Alps	-0.0783	-29.906
Central Italy	Southern Italy	Southern Alps	0.1927	94.085
Central Italy	Southern Balkan	Southern Alps	-0.0387	-13.885
Southern Italy	Northern Balkan	Southern Alps	-0.2422	-100
Southern Italy	Southern Balkan	Southern Alps	-0.2122	-79.013
Southern Balkan	Northern Balkan	Southern Alps	-0.0479	-16.372
Western Europe (N)	Central Italy	Southern Alps	-0.0274	-6.141
Western Europe (N)	Northern Balkan	Southern Alps	-0.1021	-26.528
Western Europe (N)	Southern Italy	Southern Alps	0.1463	36.376
Western Europe (N)	Southern Balkan	Southern Alps	-0.0668	-16.827
Western Europe (S)	Central Italy	Southern Alps	-0.0147	-3.089
Western Europe (S)	Northern Balkan	Southern Alps	-0.0907	-21.724
Western Europe (S)	Southern Italy	Southern Alps	0.1601	35.693
Western Europe (S)	Southern Balkan	Southern Alps	-0.0543	-12.955
Western Europe (S)	Western Europe (N)	Southern Alps	0.0205	5.708
Central Italy	Northern Balkan	Southern Italy	0.1074	52.96
Central Italy	Southern Balkan	Southern Italy	0.1071	41.563
Southern Balkan	Northern Balkan	Southern Italy	-0.0033	-1.27
Western Europe (N)	Central Italy	Southern Italy	-0.1806	-53.737
Western Europe (N)	Northern Balkan	Southern Italy	-0.09	-23.136
Western Europe (N)	Southern Balkan	Southern Italy	-0.089	-23.198
Western Europe (S)	Central Italy	Southern Italy	-0.1666	-47.366
Western Europe (S)	Northern Balkan	Southern Italy	-0.0727	-17.622
Western Europe (S)	Southern Balkan	Southern Italy	-0.0717	-17.425
Western Europe (S)	Western Europe (N)	Southern Italy	0.0276	8.129
Western Europe (S)	Western Europe (N)	Southern Balkan	0.0399	9.492
Southern Balkan	Northern Balkan	Western Europe (N)	0.0298	7.692
Southern Balkan	Northern Balkan	Western Europe (S)	0.0316	7.471

Topology for D statistics is (W, X, Y, O), in which the O is the constant ougroup *A. bedriagae*.

Supplementary table S5 Model and parameters of ancestral range estimation of extant *Podarcis muralis* lineages

Model	lnL	nParam	d	e	AICc	w
DEC	-27.55	2	1.29	3.77	60.09	4.5×10^{-5}
DIVALIKE	-22.84	2	1.21	10^{-12}	50.69	0.0049
BAYAREALIKE	-41.02	2	1.64	5	87.03	6.3×10^{-11}

lnL: log-likelihood; d: rate of range expansion; e: rate of range reduction through extirpation in an area; AICc: Akaike information Criterion adjusted for small samples; w: Akaike weights.

Supplementary table S6 Permits for specimen collection

Permit ID	Issuing authority
No. 6584	Ministry of Environment of Albania
141911/1567/26-5-2016 and 73ΟΣ4653Π8-7Ψ8	Ministry of Environment and Energy of Greece
ref2017-01666 and #2012-10	Direction Départementale de la Protection des Populations of France
DPN-2009-0005106, PNM-2012-0009747 and PNM-2017-0008287	Ministry of Environment of Italy
No. 9303/2009-2.1/jam and 4145/2011-2.2	Ministry of the Environment of the Slovak Republic
35601-66/2013-4	Ministry of Agriculture and the Environment of Slovenia
OAEN/SVSIA/avp_10_169_aut	Organismo Autónomo Espacios Naturales de Castilla – La Mancha, Spain
EP/SA/373/2016	Delegación Territorial de Salamanca, Junta de Castilla y León, Spain
500201/24/2014/3218	Departamento de Agricultura, Ganadería y Medio Ambiente, Gobierno de Aragón, Spain
B.30.2.DEU.O.A8.00.00/54	Turkish Environmental Authority
Presidential Decree 67/81*	President of Greece

* issued to the Natural History Museum of Crete

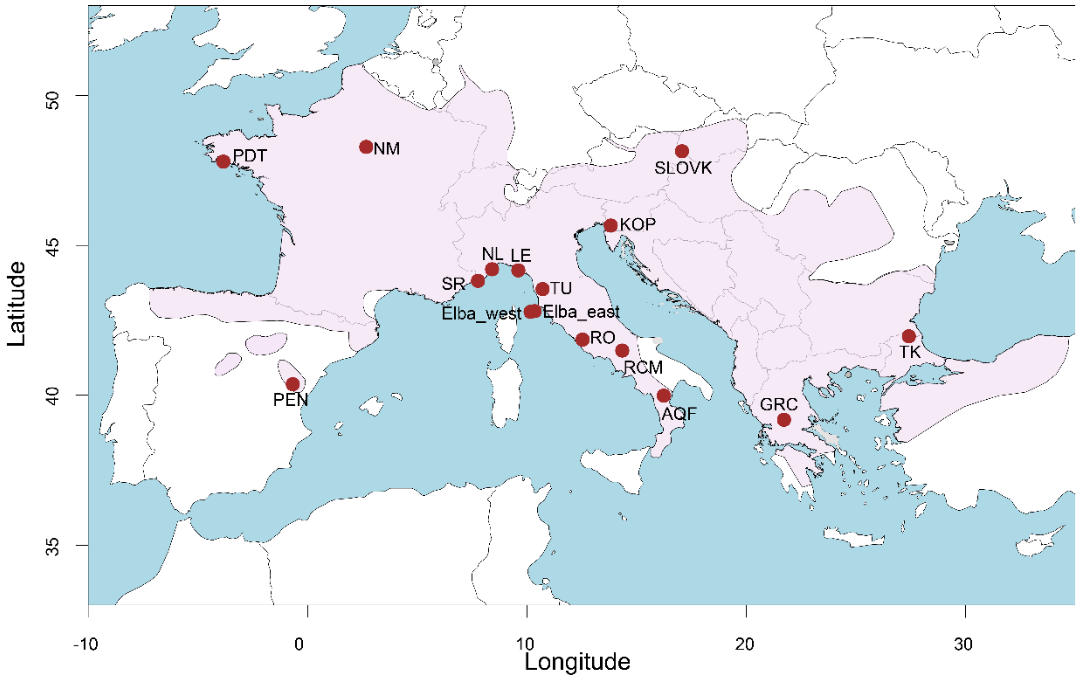

Supplementary fig. S1 Collection sites for samples used for whole genome sequencing in this study. The pink area represents the distribution range of *P. muralis*.

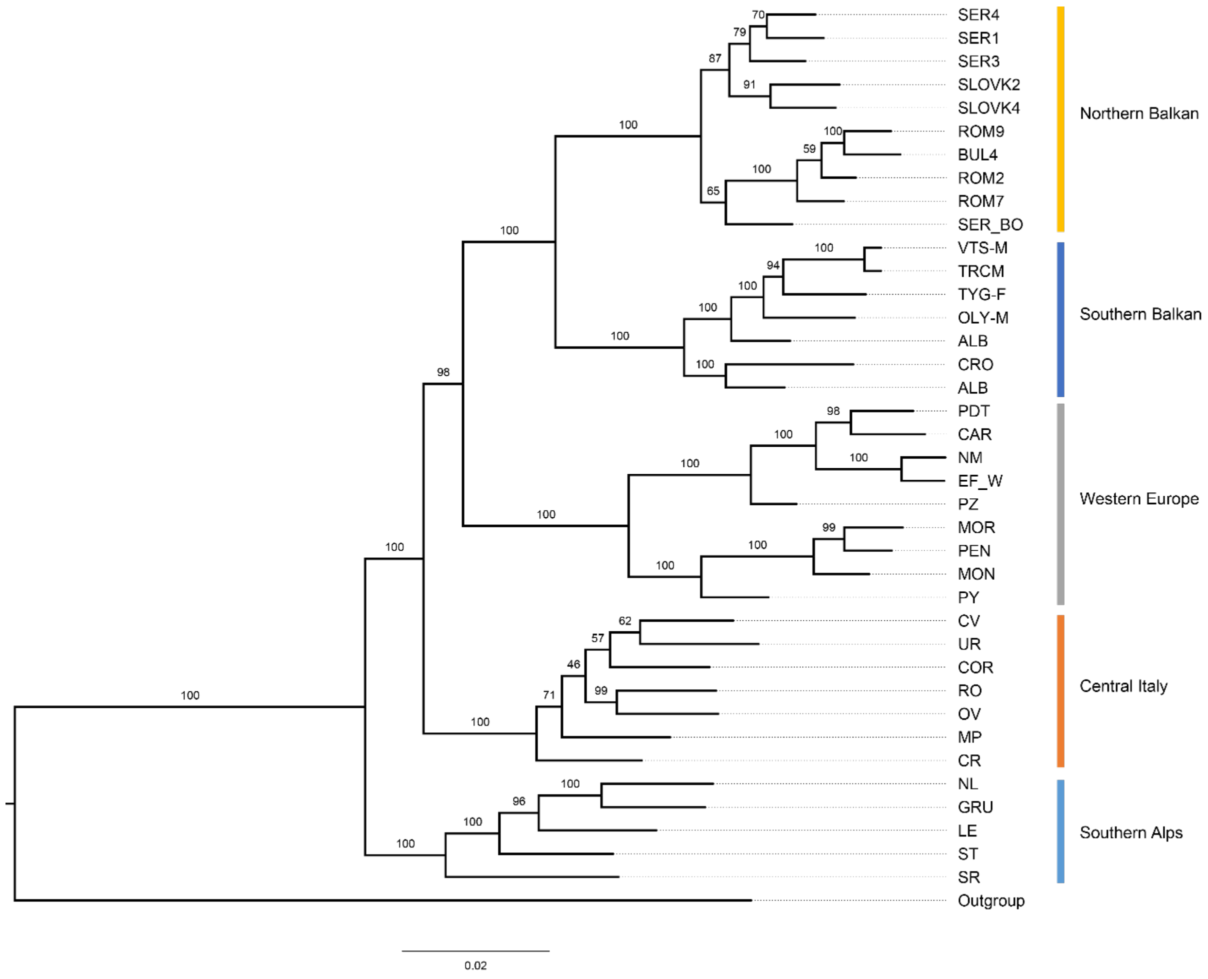

Supplementary fig. S2 Maximum likelihood phylogeny inferred by RAD-Seq data without the Southern Italy lineage. The numbers above branches indicate the bootstrap support values.

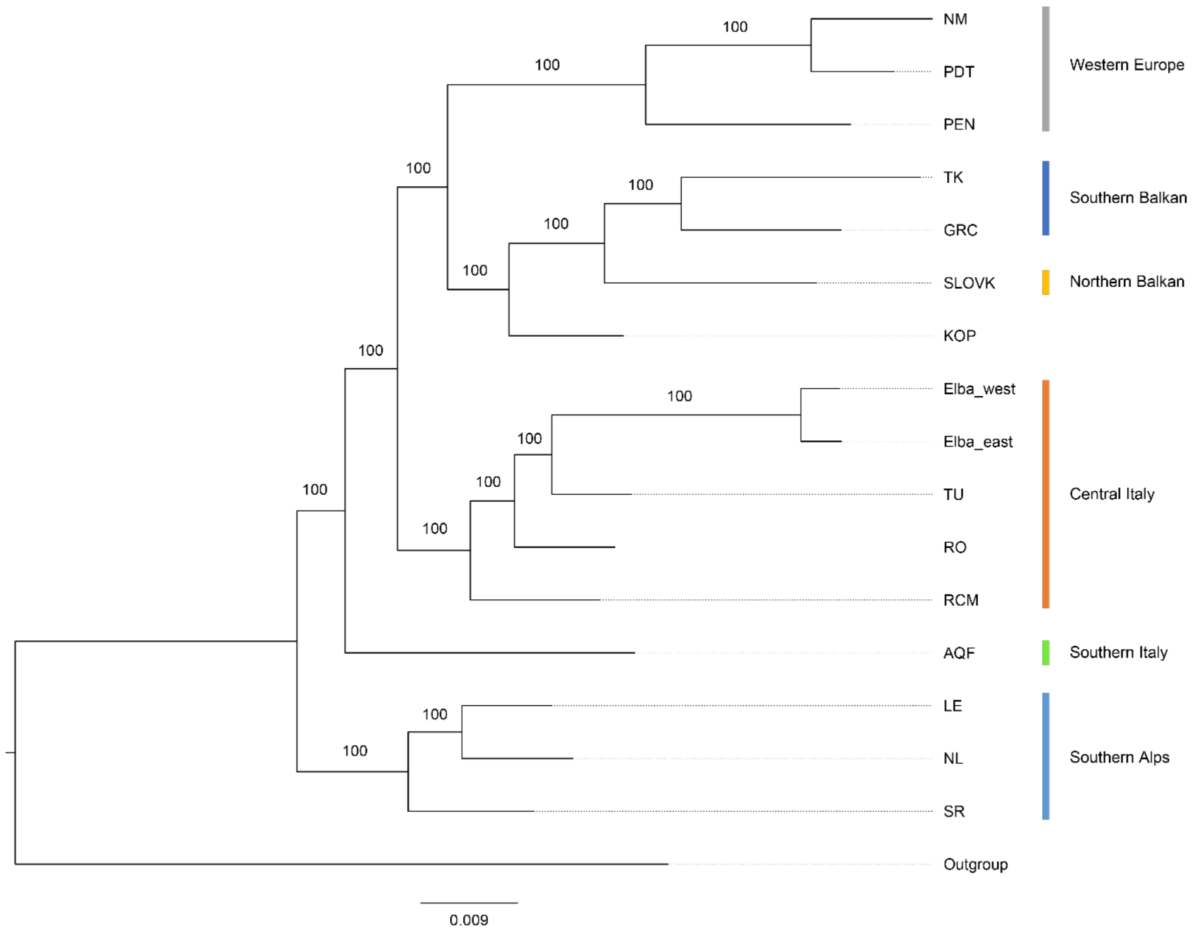

Supplementary fig. S3 Maximum likelihood phylogeny inferred by WGS data for all 16 samples. The numbers above branches indicate the bootstrap support values. The admixed individual from KOP was sister to the Balkan clades.

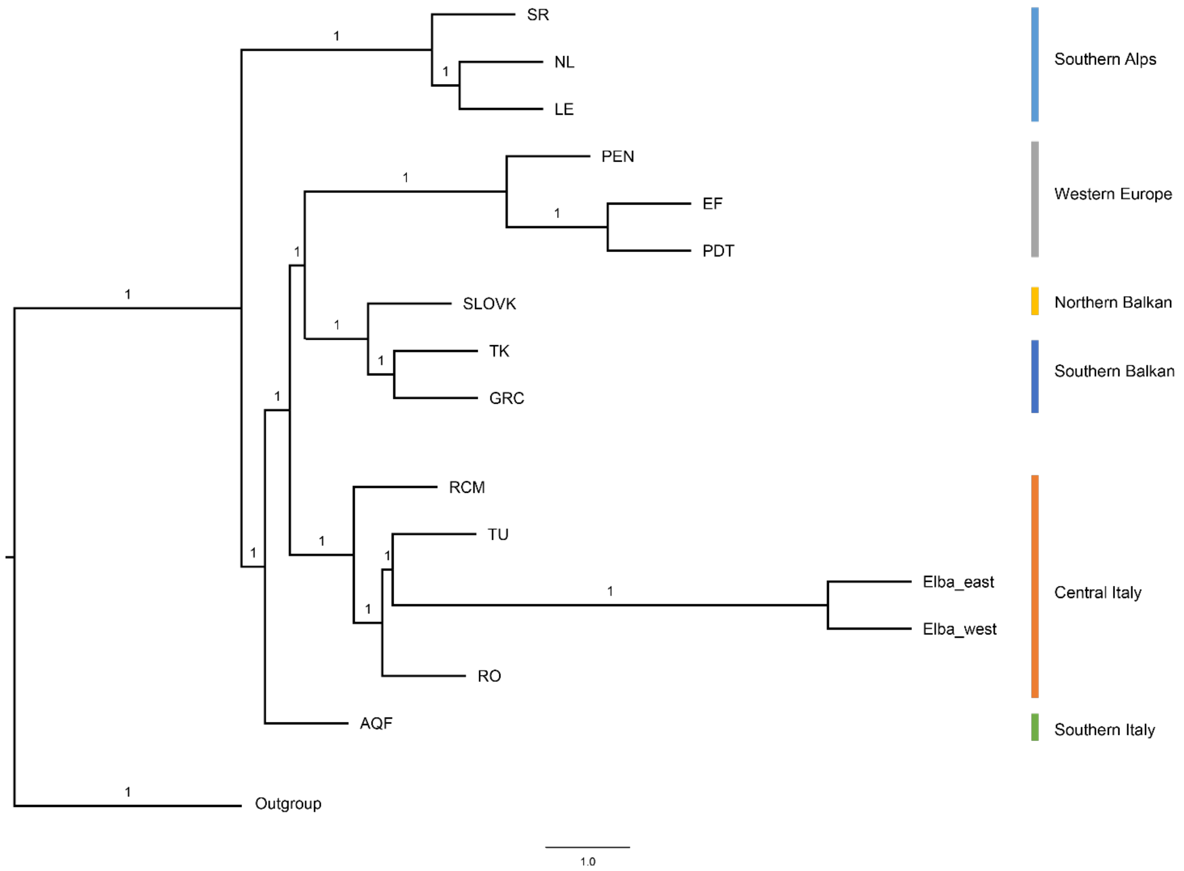

Supplementary fig. S4 Phylogeny inferred by multispecies coalescent approach for whole genome sequencing data. The numbers above branches indicate the bootstrap values for genetic lineages.

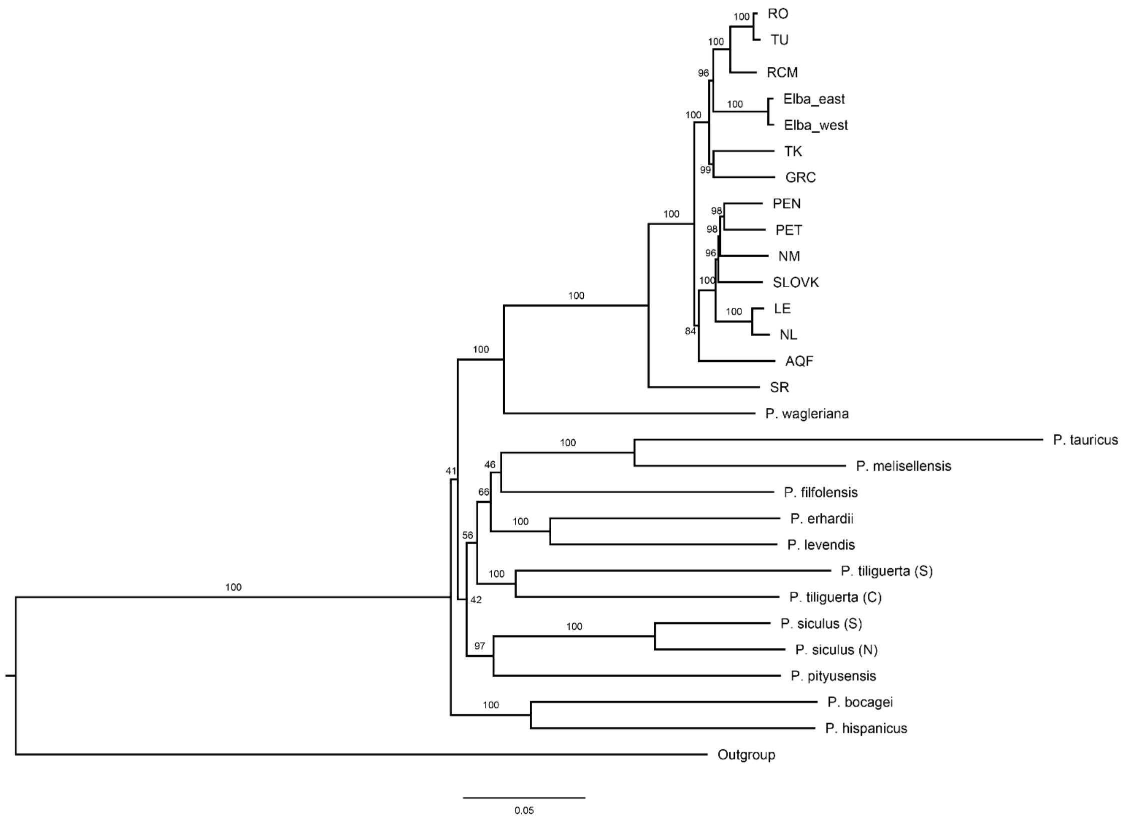

Supplementary fig. S5 The phylogeny inferred by mitochondrial genome data with *Podarcis* samples. The numbers above branches indicate the bootstrap values for genetic lineages.

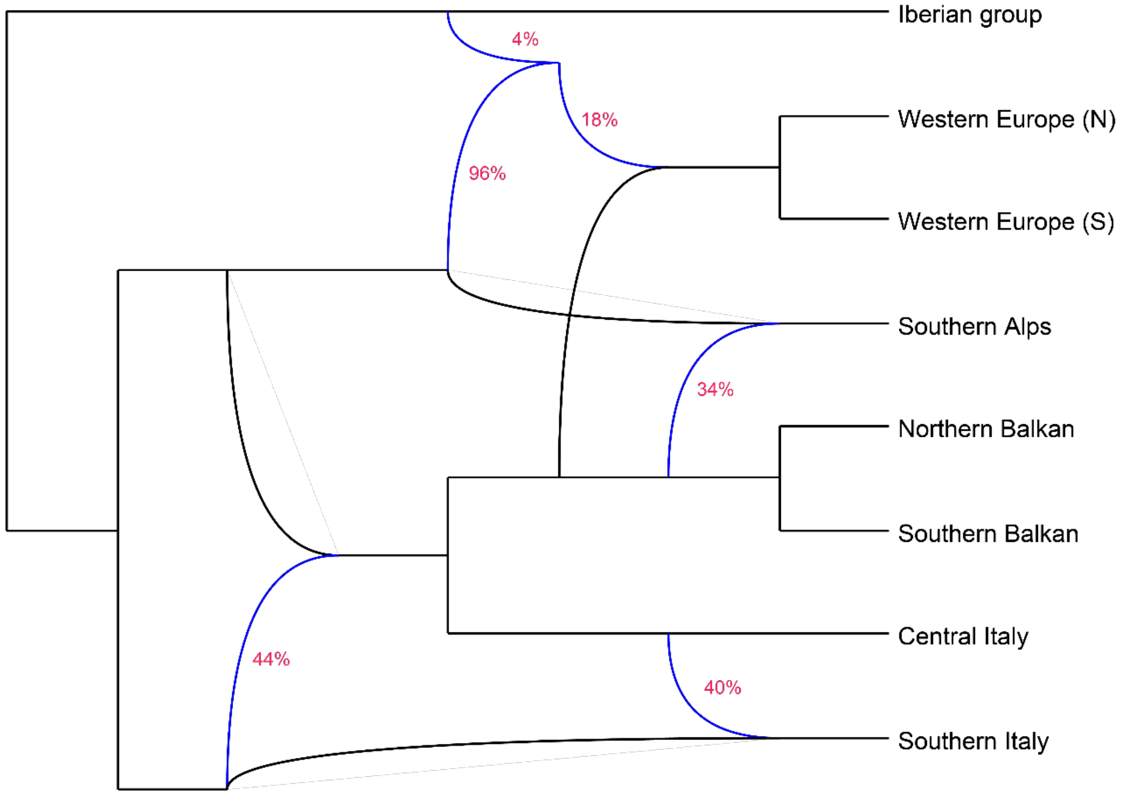

Supplementary fig. S6 Phylogenetic network inferred by phyloNet. The blue lines represent the reticulation events, and the red numbers represent the proportion of alleles from parental lineages.

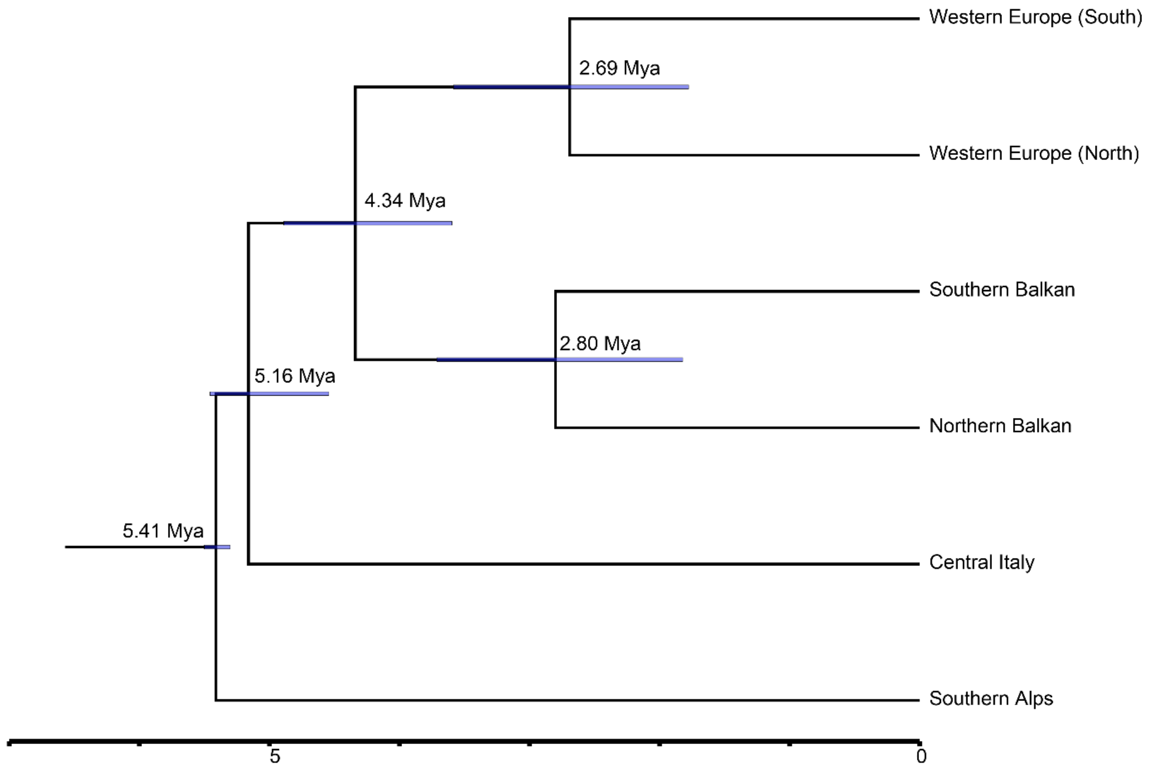

Supplementary fig. S7 Time-calibrated phylogeny for *P. muralis* without the Southern Italy lineage. The numbers indicate the estimated ages for each node. The blue bars represent the confidence interval of nodes.

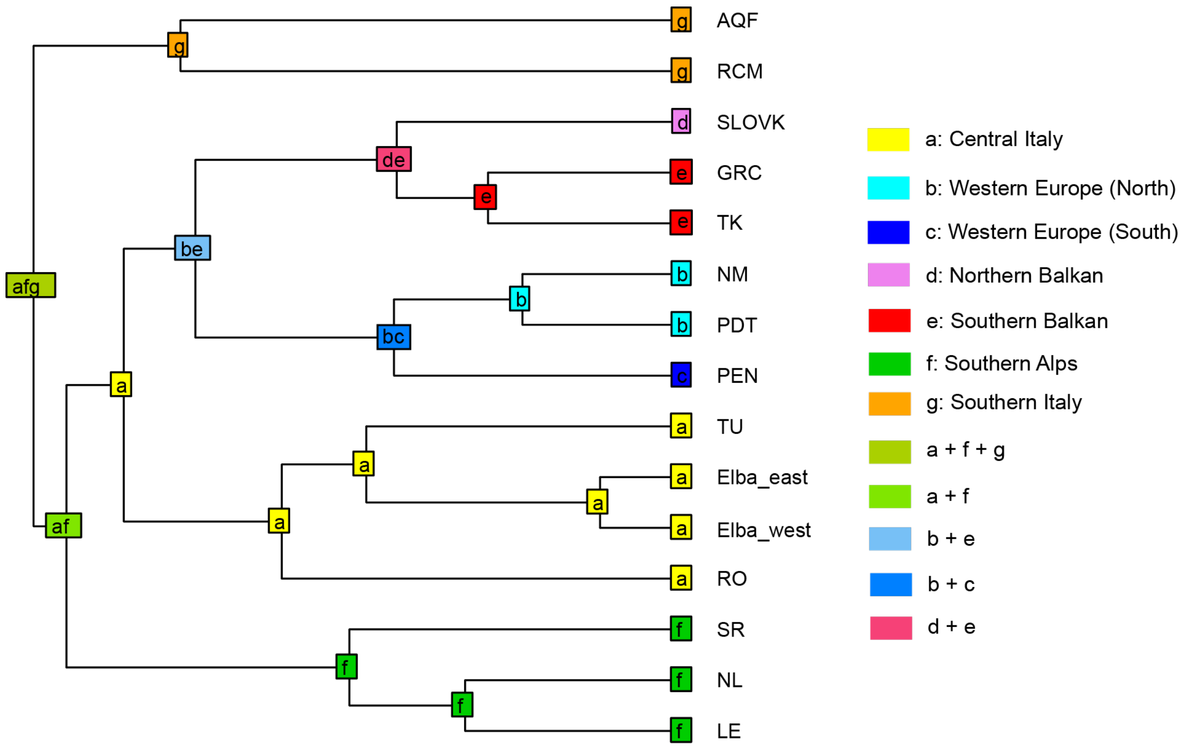

Supplementary fig. S8 The best-fitting model (DEC) in BioGeoBEARS analysis for *P. muralis* lineages based on WGS data. The colored squares represent the distributed area.
