# Supplementary material for: Population Genomics of Wall Lizards Reflects the Dynamic History of the Mediterranean Basin
Source: Mol Biol Evol. 2021 Oct 28;39(1):msab311. doi: 10.1093/molbev/msab311 (PMC8760935; doi:10.1093/molbev/msab311)
Supplement: msab311_Supplementary_Data [file msab311_supplementary_data.zip › Response to reviewers.docx]

Dear Editor,

Thank you very much for your decision. We appreciate the positive and constructive comments that we received from you, the Associate Editor and reviewer. And we took this opportunity to improve the quality of this manuscript. Please find below the report of the manuscript revision. For your convenience, we show parts newly inserted into the manuscript as underlined. Page and line numbers refer to positions in the document with tracked changes.

On behalf of all co-authors,

Dr. Weizhao Yang

Lund University

Editors’ comments to the author:

The comments and recommendations from one expert reviewer are now available for your manuscript. The associate editor served as the second reviewer and agreed with the sentiment of the other reviewer. These two reviewers judged the reported discoveries to be of high significance, and the potential scientific impact of your work to be high. They found that the manuscript needs some clarification in text.

**RESPONSE:** Thank you very much for the positive evaluation of our work. We appreciate that the editor and reviewers found the manuscript sufficiently significant to warrant publication in *Molecular Biology and Evolution*. Detailed responses to the reviewer’s comments are found below. We thank you for your time and effort in handling our manuscript.

Reviewer Comments to the Author

Yang and colleagues use the well-known wall lizard system to conduct ‘next-generation phylogeography’ in the truest sense of the word. Data includes both RADseq and whole genome sequencing. These are used to look at population structure, dated phylogeny (including extensive mt-nu DNA discordance), gene flow between the lineages, ancestral range reconstruction and demographic history inference. Italy is inferred as the most likely place of origin for the most recent common ancestor for the taxon and post-Messinian (pre-Pleistocene) colonization of the Mediterranean Peninsulas gave rise to contemporary geographical populations/lineages.

I struggle to find points of criticism. As far as I can see the extensive analyses were conducted properly and together make for a very complete story. The writing is clear and I simply like this MS. So let me just close with some minor issues in the discussion:

**RESPONSE**: We are grateful for the strong endorsement and appreciation of our study.

Line 311-313: This sentence is a bit too brief, it is not clear how dating from German fossils get you to this interpretation. I don’t know the literature and this is likely the case for most if not all readers.

**RESPONSE**: We agree that this warrants some more information. We cited the fossils here to support the argument that *P. muralis* may have migrated out of Italy following the Messinian salinity crisis, which has been clarified in lines 312 – 315.

**Originally:** *The descendants of this ancient Italian assemblage appear to have followed an “Out-of-Italy” dispersal route to the Balkan and Iberian Peninsulas after the Messinian salinity crisis, a pattern that is consistent with dating of Podarcis fossil remains from Germany (Böttcher 2007).*

**Revised:** *The descendants of this ancient Italian assemblage appear to have followed an “Out of Italy” dispersal route to the rest of the Mediterranean basin after the Messinian salinity crisis, which is consistent with the discovery of a probable P. muralis fossil from the upper Pliocene in Central Europe (Böttcher 2007; Gassert et al. 2013).*

Line 330: Positive selection must drive part of it, but I guess it is not the only part of the story. What about selectively neutral stuff? There must have been many opportunities for regaining of secondary contact with potential hybrid zone movement and neutral introgression in the system. Should the possibility not at least be mentioned?

**RESPONSE**: We agree that it is an important point. Thank you very much. There is no direct evidence in *P. muralis* for hybrid zone movement, but its importance and consequences for patterns of neutral introgression have been well studied in other organisms. We have added this possibility and one additional reference in lines 332-334.

**Originally:** *... which demonstrate that significant parts of a genome can introgress under positive selection (While et al. 2015; Yang et al. 2018; see also Schulte et al. 2012; Beninde et al. 2018).*

**Revised:** *... which demonstrate that significant parts of a genome can introgress under positive selection, with possible additional contribution by hybrid zone movement and neutral introgression (While et al. 2015; Yang et al. 2018; see also Schulte et al. 2012; Beninde et al. 2018; Wielstra 2019).*

Line 408: related to above, I am not sure how this follows, the citations refer to only one hybrid zone but why would this apply to all hybrid zones?

**RESPONSE:** Thank you for picking up this ambiguity. We have modified this sentence to avoid over-interpretation of the current studies on the hybrid zone of *P. muralis* in lines 410-413.

**Originally:** *... and therefore contributed to the persistence of lineages following secondary contact, and explain the location and apparent stability of contemporary hybrid zones (e.g., between the SA and CI lineages; Yang et al., 2018, 2020).*

**Revised:** *... and therefore contributed to the persistence of lineages following secondary contact. For example, this scenario may explain the apparent stability of the contemporary hybrid zone between the SA and CI lineages (Yang et al. 2018, 2020).*
